# Idiopathic Bilateral Ovarian Vein Thrombosis in a Non-Pregnant Healthy Patient: A Case Report and Review of the Literature

**DOI:** 10.7759/cureus.10111

**Published:** 2020-08-29

**Authors:** Abdul Basit, Pushpinder Kaur, Diana M Villanueva, Muhammad Tahir, Mark Sonnenschine

**Affiliations:** 1 Internal Medicine, Coney Island Hospital, Brooklyn, USA; 2 Pathology, Case Western Reserve University School of Medicine, Cleveland, USA; 3 Hematology, Coney Island Hospital, Brooklyn, USA

**Keywords:** ovarian vein thrombosis, bilateral thrombosis, idiopathic ovarian vein thrombosis

## Abstract

Ovarian vein thrombosis (OVT) is a potentially life-threatening condition, and it is typically related to the peripartum period; however, it is also associated with pelvic inflammatory disease, recent pelvic or abdominal surgery, inflammatory bowel disease, thrombophilia, malignancy, and sepsis. Idiopathic isolated OVT is rare and is usually presented as case reports in the medical literature. In this report, we present a case of bilateral OVT in a postmenopausal female with no identifiable risk factors and normal coagulation profile workup to highlight the importance of considering it as a differential diagnosis in female patients presenting with abdominal pain. Early identification can prevent potentially life-threatening complications. Management is often conservative, and the choice of anticoagulation is based on the patient’s medical conditions. In this particular scenario, the patient was managed with low molecular weight heparin (LMWH) and transitioned to direct oral anticoagulant (DOAC) before discharge.

## Introduction

The first case of ovarian vein thrombosis (OVT) was reported in 1956 by Trang et al. [1]. OVT is mainly associated with postpartum complications. Other associated etiologies include pelvic or abdominal surgeries, pelvic infection, sepsis, trauma, malignancy, and hypercoagulable states. The incidence rate increases with cesarean delivery, and untreated thrombosis can cause pulmonary embolism, increasing mortality up to 4% [2]. The most common presenting complaint of OVT is abdominal pain associated with gastrointestinal symptoms. Early diagnosis is very important; abdomen and pelvic CT with intravenous contrast remains the main choice of imaging to successfully differentiate it from and rule out other pathological diseases related to female pelvic anatomy. The treatment of choice is anticoagulation, and the use of antibiotics is also indicated if signs and symptoms of pelvic infection are present [1,2].

## Case presentation

Our patient was a 41-year-old African American woman, G6P6006 (all non-complicated vaginal deliveries and last delivery had been seven years ago). Her past medical history was unremarkable except for the chronic pelvic pain that she had been experiencing for the past five years. She presented to the emergency department complaining of sudden onset of worsening abdominal pain in the bilateral iliac fossa region radiating to the pubic symphysis. The pain was sharp and constant, associated with nausea and constipation. Her last menstrual period had been two years ago, and there had been no recent or remote history of contraceptive use, hematuria, trauma, prolonged traveling, surgeries, or infection. Upon questioning, there was no significant family history of any hematological disorders.

On physical examination, her oral temperature was 36.6 °C; she had a heart rate of 67 bpm, respiratory rate of 20 bpm, blood pressure of 132/77 mmHg, and oxygen saturation of 99% on room air. The patient was alert and oriented with remarkable findings of the left and right lower quadrant tenderness that was appreciated on palpation without the signs of guarding. The remaining systemic and neurological examinations were unremarkable. No abnormalities were reported on initial clinical laboratory investigations. Prothrombin time (PT), international normalized ratio (INR), and activated partial thromboplastin time (aPTT) were within normal limits. Since the patient initially presented with abdominal pain, hypercoagulable studies were not ordered. Later on, all hypercoagulability workups including but not limited to antithrombin III, protein C and S, and all the clotting factors were within normal limits. The patient was also evaluated for pelvic infections and complete blood count (CBC), erythrocytes sedimentation rate (ESR), and C-reactive protein (CPR) along with pro-calcitonin, which all came back normal. Gynecological consultation was ordered to find out the existence of any cervical or uterine pathologies and all the possible causes of pelvic infection were ruled out.

**Figure 1:**
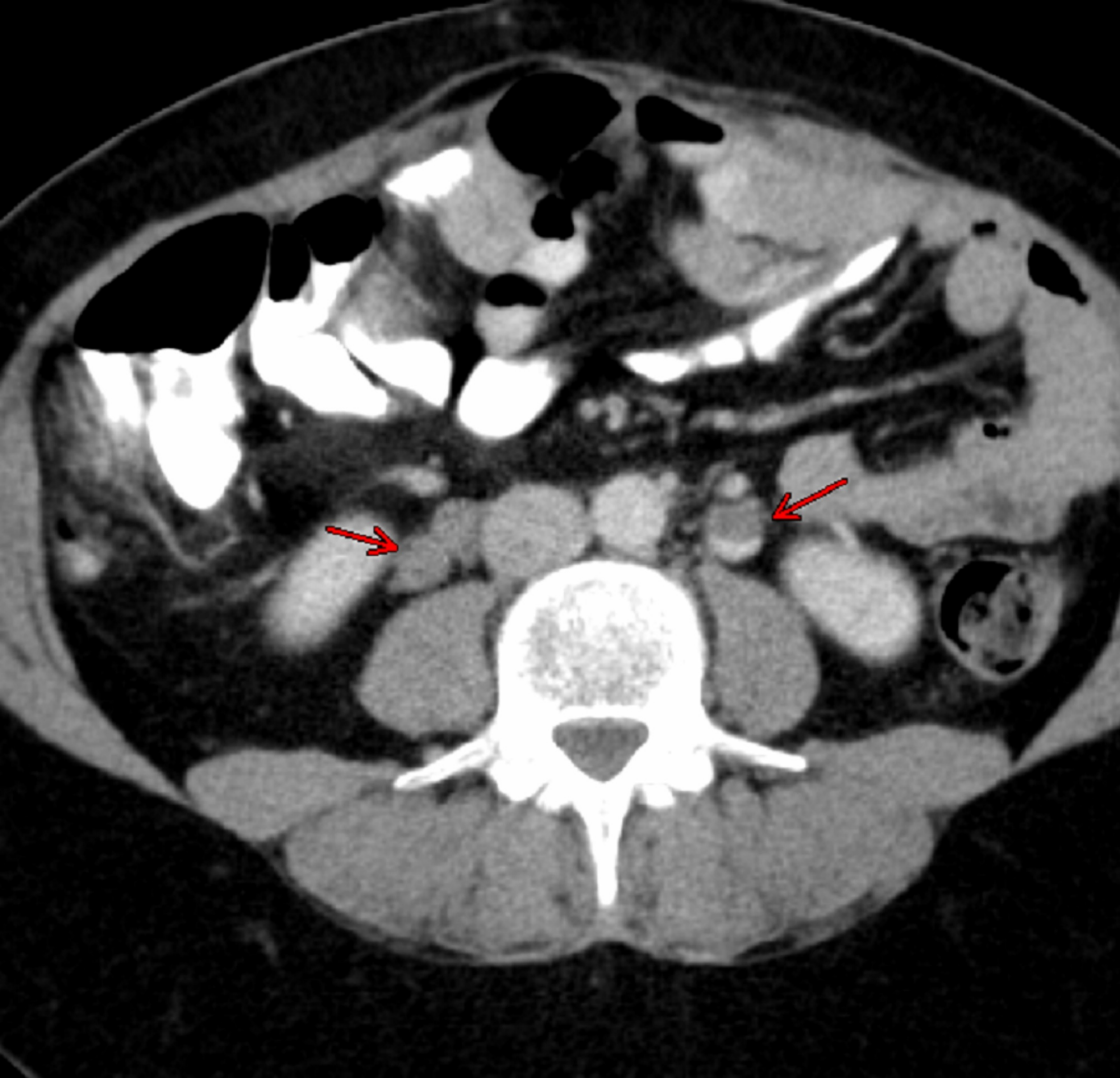
CT image showing filling defect in the right and left gonadal vein (red arrows) CT: computed tomography

**Figure 2:**
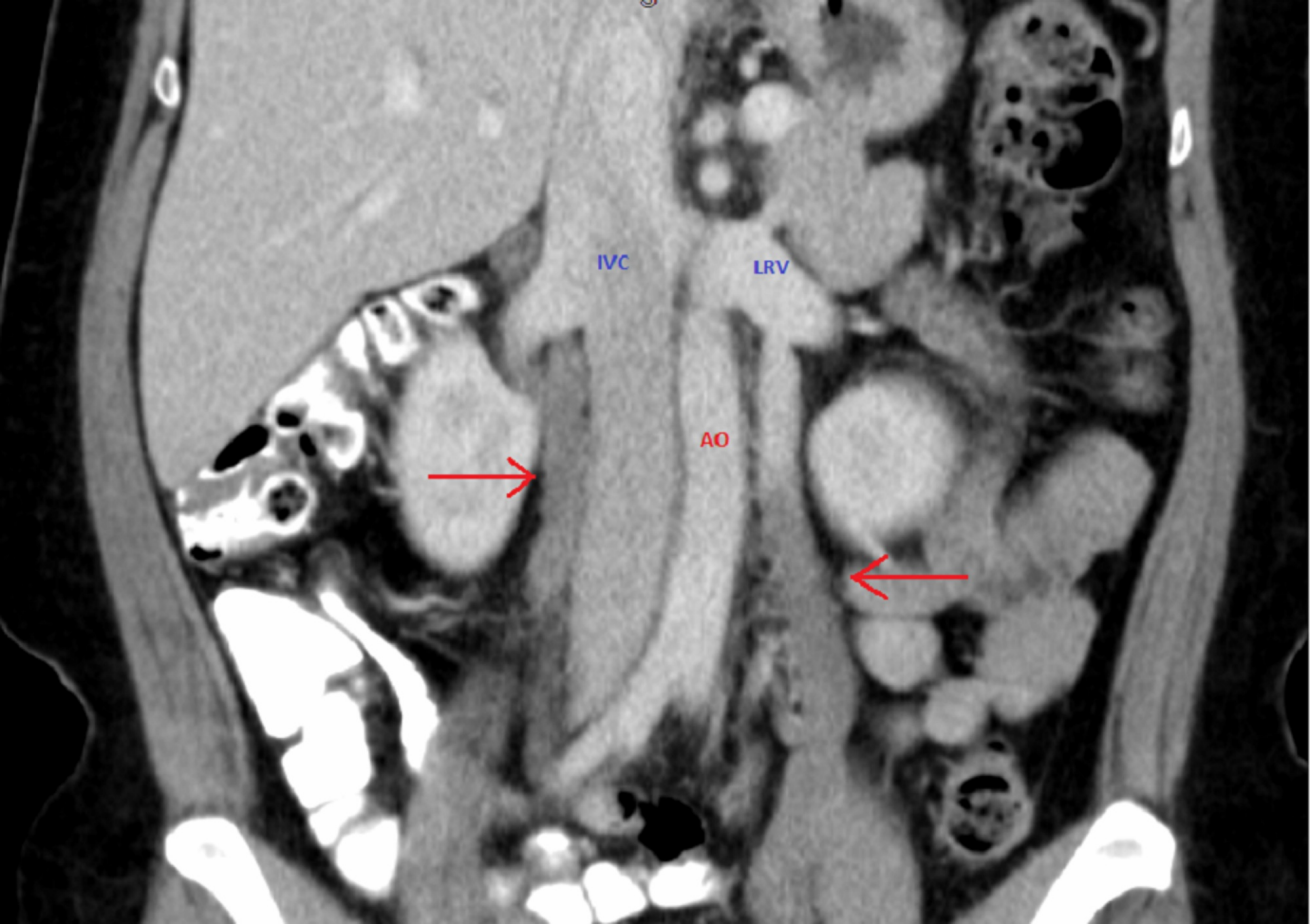
CT of the abdomen (coronal section) showing bilateral OVT as indicated by the red arrows CT: computed tomography; OVT: ovarian vein thrombosis; AO: abdominal aorta; IVC: inferior vena cava; LRV: left renal vein

Abdominal and pelvic CT with oral and intravenous contrast revealed an apparent filling defect in the right and left gonadal veins, as shown in Figure 1. Another CT of the abdomen coronal revealed bilateral OVT as shown in Figure 2. Therefore, the diagnosis of bilateral OVT was made, and blood samples were obtained for coagulation profile and the patient was started on therapeutic low molecular weight heparin (LMWH) followed by direct oral anticoagulant (DOAC). The patient’s abdominal pain significantly improved by the third day of starting the anticoagulation, and she was discharged home with oral anticoagulation on apixaban. The patient is scheduled for follow-up at three- and six-month intervals, and the plan is to complete six months of anticoagulation therapy provided the thrombosis is resolved at the end of the treatment period.

## Discussion

The incidence of OVT ranges from 0.05% to 0.18% in normal vaginal deliveries but it goes up to 2% with cesarean deliveries and twin pregnancies [2-5], with 90% of the cases involving right-sided ovarian vein [2]. OVT is a rare yet potentially fatal condition with a 52% mortality rate if left untreated [3]. It can extend to inferior vena cava or renal veins due to anatomical proximity. According to one report, the incidence of pulmonary embolism arising from OVT is 33%, with ~4% mortality rate. Other complications include sepsis and chronic pelvic pain [3,6,7]. The classical Virchow’s triad can explain the pathophysiology and its increased incidence associated with pregnancy. During pregnancy, the blood flow in ovarian veins increases significantly, and the venous valves become incompetent. At the same time, the sudden change after childbirth causes venous collapse and stasis [3]. The possible etiologies for underlying causes for increased right-sided OVT are related to its longer course, more incompetent valve, and direct pressure from the gravid uterus [8]. Hypercoagulability also increases during pregnancy, sepsis, and malignancy or in vivo hypercoagulable state, leading to thrombosis. The surgical procedure causes intimal injury of endothelium and hence prone to thrombosis.

Idiopathic OVT is very rare, and no causative factor has been identified. The first case of idiopathic OVT was reported in 2004 by Yildirim et al. [6]. Since then, 12 cases of unilateral idiopathic OVT have been reported. Upon careful review of these reported cases, we observed that few had risk factors that may lead to OVT. In one of the reported cases, the patient had polycystic ovarian syndrome (PCOS) and recent use of oral contraceptive (OCP), which had been discontinued eight weeks before the presentation [9], while in another reported case, the patient had a recent history of pelvic inflammatory disease with evident bilateral hydrosalpinx and had undergone bilateral laparoscopic salpingectomy three weeks before the presentation, contributing to significant risk factors [10]. One more patient had experienced recent significant trauma, and even at the time of presentation with OVT, had healing bruises at the abdominal wall [5].

Among the reported cases, ~65% had right-sided isolated OVT, including one with a 3-cm mass [11]. None of them had a fever at presentation, while only a few had mild leukocytosis. Otherwise, the initial lab workup remained normal. Abdominal pain was consistently present in all the reported cases and almost always indicated the laterality of the OVT. Furthermore, most of the cases were acute to subacute. However, in the previously reported case of bilateral thrombosis [4] and our presented case, patients had a long-standing history of abdominal pain, going back to several years, and presentation was characterized by acute over chronic abdominal pain.

To the best of our knowledge, only one case of idiopathic bilateral ovarian thrombosis has been reported previously [4]. Moreover, in the previously reported case, the patient had a medical history of bilateral pulmonary embolism five years prior to presenting with bilateral OVT, which was one week after childbirth, and also had several inconclusive CT angiograms of the chest performed because of chest pain and elevated D dimers. Our case was unique as the patient had no previous medical or family history of any previous thrombosis, no recent history of sepsis, hospitalization, traveling, oral contraceptives, trauma, surgery or pelvis infection, or any remote history of unexplained abortion. The patient had chronic bilateral flank pain, similar to the previously reported case, for almost three years. All hypercoagulability workups remained negative, including beta 2 glycoprotein 1 immunoglobulin G (IgG), IgA, IgM antibodies, factor IX, V, VIII, XI, XII assay, factor V Leiden mutation, activated protein C (APC) resistance, AT III activity/antigen, D dimers, fibrinogen levels, hexagonal phase lupus assay, lupus anticoagulant panel including anticardiolipin, methylmalonic acid, homocysteine levels, plasmin F levels, protein C and S antigen, thrombin time, platelets number, bleeding time, and prothrombin time (INR). Furthermore, after an extensive examination of the history and workup, the patient was deemed to have idiopathic bilateral OVT.

Different imaging modalities can make the diagnosis of OVT. Doppler ultrasound (DUS) remains the first choice, as it is non-invasive, inexpensive, and readily available in the emergency department. However, it is operator-dependent, and body habitus, overlying structures, and bowel gas patterns can interfere with imaging [4,5]. The sensitivity of the DUS is around ~50% only [5]. Also, DUS cannot examine the whole length of the ovarian veins [2]. Hence, further imaging modality is warranted. CT venogram or CT abdomen pelvis with contrast is also sensitive and specific for the diagnosis and is time- and cost-effective. Magnetic resonance angiography (MRA) remains the gold standard but is expensive and not widely available. In all the reported cases, almost all the patients were successfully diagnosed with abdomen and pelvis CT with intravenous contrast, making it the imaging of choice for the diagnosis of OVT.

Management of OVT is mainly conservative with anticoagulation and antibiotics indicated only if the pelvic infection is present. Surgical interventions, including the insertion of an inferior vena cava filter or ovarian veins ligation, might be warranted if there is evidence of persistent thrombosis with a high risk of complications and anticoagulation failure, or if a contraindication to anticoagulation is present. Among all the reported cases of OVT, the majority were treated initially with unfractionated heparin, with warfarin being added later on. No complications or failure of anticoagulation was reported. Furthermore, serial follow-ups showed the resolution of OVT. Only one case was managed by DOAC (rivaroxaban) [4], with complete resolution of bilateral OVT at six weeks. In our case, the patient was initially started on therapeutic LMWH and later switched to DOAC (apixaban).

No guidelines are available regarding the management of OVT. However, multiple authors [2,4] have argued that the lower-limb deep vein thrombosis (DVT) guidelines are applicable in this scenario. The duration of treatment and choice of anticoagulation remains an essential aspect of management. The duration of the anticoagulation is typically three to six months for transient or provoked conditions. In comparison, lifelong anticoagulation might be needed for persistent or hypercoagulable states. Since the introduction of DOAC, these agents have become a widely used method of anticoagulation. However, their safety during childbearing age is concerning. Animal studies have shown increased fetal toxicities, and no human studies are available. Nevertheless, regarding DOAC, the summary of product characteristics (SPCs) recommends against its usage during pregnancy and lactation. Warfarin is also contraindicated during pregnancy, and proper contraception use is mandatory for usage during childbearing age. LMWH is an alternative if pregnancy occurs, and DOAC or warfarin should be discontinued immediately [12]. The studies included for literature review were collected from PubMed and are listed below in Table 1.

**Table 1:** Summary of included studies of ovarian vein thrombosis with details of signs, symptoms, diagnostic and therapeutic interventions, and complications and follow-ups DOAC: direct oral anticoagulant; PMHx: past medical history; CT: computed tomography; US: ultrasound; PCOS: polycystic ovarian syndrome; OCPs: oral contraceptive pills; IV: intravenous

No.	Author	Age at the time of presentation (years)	Duration of pelvic pain with constitutional signs and symptoms	Imaging modality used for diagnosis	Ovary with thrombus	Treatment	Complications/follow-up
1	Trang et al. [1]	47	1 day of acute back pain radiating to the abdomen with dyspnea	CT abdomen and pelvis with IV contrast	Left	Low molecular weight heparin, followed by DOAC (rivaroxaban) for 3 months	Follow-up at 2 months showed complete resolution of thrombus
2	Alalqam et al. [2]	42	1 day of acute left iliac fossa pain, nausea, constipation with abdominal tenderness and guarding	US abdomen, Doppler US, CT abdomen and pelvis with IV contrast	Left	Low molecular weight heparin, followed by warfarin for 6 months	Follow-up at 1 year showed complete resolution of thrombus
3	Garcia et al. [4]	35	2 days of worsening left flank pain. History of low intensity left flank pain for 5 years. PMHx: bilateral pulmonary embolism 5 years ago, several non-diagnostic CT chests with elevated D dimer levels	CT abdomen and pelvis with IV contrast	Bilateral ovarian vein thrombosis	DOACs (rivaroxaban) for 3 months	Follow-up US at 6 and 12 weeks showed complete resolution of thrombus
4	Kodali et al. [5]	40	1 day of acute abdominal pain, with nausea. PMHx: significant abdominal wall trauma 2 months prior to presentation	CT abdomen and pelvis with IV contrast	Right	Low molecular weight heparin followed by warfarin for 6 months	No follow-up/complication details were provided
5	Yildirim et al. [6]	36	Peritoneal signs were present for 2 days	US, Doppler US, then CT abdomen pelvis with contrast	Right	Unfractionated heparin, followed by warfarin for 6 months	Calcification of thrombus was identified after 6 months of presentation
6	Stafford et al. [7]	42	1 day of acute abdominal pain with nausea, with local rebound tenderness	CT abdomen pelvis with contrast	Right	Unfractionated heparin, followed by warfarin	Follow-up US at 2 months showed complete resolution of thrombus
7	Doherty et al. [8]	29	8 months of acute over chronic abdominal pain	Doppler US, CT abdomen and pelvis with IV contrast	Left	Warfarin therapy for 6 months	No evidence of thrombus was found on follow-up at 2 months
8	Murphy et al. [9]	27	2 week’s subacute abdominal pain with low appetite and nausea, vomiting. PMHx: PCOS treated with OCPs, self-discontinuation 2 months prior to presentation. Pt had one copy of the C677T mutation of the MTHFR gene with no A1298C mutation	US abdomen was negative while CT with oral and IV contrast concluded the diagnosis	Right	No details provided	No follow-up/complication details were provided
9	Heavrin et al. [10]	29	3 days of acute abdominal pain, and tenderness with voluntary guarding, nausea, and vomiting. PMHx: 3 weeks earlier diagnosed with PID, and bilateral hydrosalpinx. Underwent D&C, and bilateral laparoscopic salpingectomy	CT abdomen and pelvis with contrast	Left	Initially, the patient left against medical advice and presented 1 week later with chest pain; diagnosed with pulmonary embolism by CT chest with IV contrast. 6 months of anticoagulation with no details about the choice of anticoagulant	No complications at 18-month follow-up and no recurrence with complete resolution of the previous thrombosis
10	Harris et al. [11]	53	1 week of subacute abdominal pain with a 3-cm palpable mass in the right lower quadrant	CT abdomen and pelvis with IV contrast	Right	Warfarin for 5 months	CT showed persistence of thrombosis with no further extension beyond ovarian veins
11	Chebl et al. [13]	79	7 days of subacute abdominal pain	CT abdomen and pelvis with IV contrast	Right	Warfarin for 3-6 months	No follow-up/complication details were provided
12	Khishfe et al. [14]	30	1 day of acute abdominal pain with nausea and local tenderness	CT abdomen and pelvis with IV contrast	Right	Warfarin and antibiotics therapy	No follow-up/complication details were provided

## Conclusions

OVT is a unique entity usually associated with the peripartum and postpartum period. Bilateral idiopathic OVT is a very rare condition that can lead to potentially fatal complications, and hence early diagnosis is very important. Hypercoagulability workup should be performed if no obvious risk factor has been identified, and anticoagulation remains the mainstay of the treatment along with antibiotics if needed. The choice of anticoagulation depends on individual risk factors, childbearing age status, and the underlying cause of OVT.
